# Fermented Milk Containing *Lacticaseibacillus rhamnosus* SNU50430 Modulates Immune Responses and Gut Microbiota in Antibiotic-Treated Mice

**DOI:** 10.4014/jmb.2401.01012

**Published:** 2024-04-30

**Authors:** Sunghyun Yoon, SungJun Park, Seong Eun Jung, Cheonghoon Lee, Woon-Ki Kim, Il-Dong Choi, GwangPyo Ko

**Affiliations:** 1 Graduate School of Public Health, Seoul National University, Seoul 08826, Republic of Korea; 2 N-Bio, Seoul National University, Seoul 08826, Republic of Korea; 3 KoBioLabs, Inc., Seoul 08826, Republic of Korea; 4 weBiom Inc., Seoul 08826, Republic of Korea; 5 R&BD Center, hy Co., Ltd., Yongin 17086, Republic of Korea; 6 Institute of Health and Environment, Seoul National University, Seoul 08826, Republic of Korea

**Keywords:** Antibiotic, fermented milk, gut microbiota, immunomodulation, *Lacticaseibacillus rhamnosus*, probiotic

## Abstract

Antibiotics are used to control infectious diseases. However, adverse effects of antibiotics, such as devastation of the gut microbiota and enhancement of the inflammatory response, have been reported. Health benefits of fermented milk are established and can be enhanced by the addition of probiotic strains. In this study, we evaluated effects of fermented milk containing *Lacticaseibacillus rhamnosus* (*L. rhamnosus*) SNUG50430 in a mouse model with antibiotic treatment. Fermented milk containing 2 × 10^5^ colony-forming units of *L. rhamnosus* SNUG50430 was administered to six week-old female BALB/c mice for 1 week. Interleukin (IL)-10 levels in colon samples were significantly increased (*P* < 0.05) compared to water-treated mice, whereas interferon-gamma (IFN-γ) and tumor necrosis factor alpha (TNF-α) were decreased, of mice treated with fermented milk containing *L. rhamnosus* SNUG50430-antibiotics-treated (FM+LR+Abx-treated) mice. Phylum Firmicutes composition in the gut was restored and the relative abundances of several bacteria, including the genera *Coprococcus* and *Lactobacillus*, were increased in FM+LR+Abx-treated mice compared to PBS+Abx-treated mice. Interestingly, abundances of genus *Coprococcus* and *Lactobacillus* were positively correlated with IL-5 and IL-10 levels (*P* < 0.05) in colon samples and negative correlated with IFN-γ and TNF-α levels in serum samples (*P* < 0.001). Acetate and butyrate were increased in mice with fermented milk and fecal microbiota of FM+LR+Abx-treated mice were highly enriched with butyrate metabolism pathway compared to water-treated mice (*P* < 0.05). Thus, fermented milk containing *L. rhamnosus* SNUG50430 was shown to ameliorate adverse health effects caused by antibiotics through modulating immune responses and the gut microbiota.

## Introduction

Antibiotics are used to control infectious diseases, but can disrupt the commensal microbiota, particularly in the intestinal tract. Antibiotics can also cause inflammatory diseases such as asthma, celiac disease, inflammatory bowel disease (IBD), and obesity [1-3] and alteration of the gut microbiota by antibiotics is positively correlated with an enhanced inflammatory response [4]. Due to the lack of effective methods to control side effects of antibiotics, various alternatives, such as antimicrobial peptides, antimicrobial enzymes, and phytochemicals have been suggested [5-7]. However, the problem of side effects of antibiotics remains to be solved.

Growing interest in the health benefits of fermented foods has resulted in increased consumption of fermented milk [8]. Probiotic strains such as *Lactobacillus delbruckii* ssp. *bulgaricus* and *Streptococcus thermophilus* are used as milk fermentation starters [9] and other probiotic strains such as *Lactobacillus* or *Bifidobacterium* spp. can be added to fermented milk to enhance its health benefits [10]. Fermented milk exerts beneficial effects on glucose and lipid metabolism disorders, including fasting blood glucose, low-density lipoprotein cholesterol, and leptin reduction, via the increasing of beneficial gut microbes, such as *Bifidobacterium* spp., and major metabolites, including docosatrienoic acid, oleanolic acid, and L-(+)-aspartic acid [11] and can modulate humoral and cellular immune responses [12]. The consumption of fermented milk has also been reported to control autoimmune diseases as a result of its anti-inflammatory effects [13, 14].

Metabolites from the gut microbiota can affect host physiology [15]. Short-chain fatty acids (SCFAs) including acetate, propionate, and butyrate, which are anaerobically fermented by-products of indigestible polysaccharides via the gut microbiota and probiotics, provide energy to gut epithelial cells and maintain intestinal mucosa [16, 17]. SCFAs control inflammation-related diseases including IBD and allergic asthma [18, 19]. The administration of *Lactobacillus* spp. can boost the production of SCFAs in the colon [20].

Therefore, in this study, we investigated effects of the fermented milk containing *Lacticaseibacillus rhamnosus* SNUG50430 in the in vivo mouse model with the antibiotics-treatment. To assess its effects on immunity and the composition of the gut microbiota, we applied fermented milk with or without *L. rhamnosus* SNUG50430 to mice with dysbiosis of the microbiota as a result of the administration of antibiotics.

## Materials and Methods

### Preparation of Fermented Milk Containing *L. rhamnosus* SNUG50430

*L. rhamnosus* SNUG50430 was isolated from feces of healthy Korean participant. We confirmed that *L. rhamnosus* SNUG50430 was resistant to harsh environmental conditions including high concentrations of bile salts and low pH (data not shown). *L. rhamnosus* SNUG50430 was cultured in anaerobic condition at 37°C for 24 h using de Man, Rogosa and Sharpe broth (BD Biosciences, USA) with 0.05% L-cysteine-hydrochloride (Sigma-Aldrich, USA). Bacterial stocks were prepared using 20% glycerol and stored at −80°C until further use.

Fermented milk containing lactic acid bacteria FD-DV8 ST-Body-1 (Chr. Hansen Holding A/S., Denmark), as the fermentation starter, was prepared in R&BD Center, hy Co., Ltd. (Republic of Korea). Subsequently, 1 × 10^6^ colony-forming units (CFUs)/ml of *L. rhamnosus* SNUG50430 were added to the fermented milk, which was stored at 4°C until use.

### In Vivo Animal Model with Antibiotic Treatment

**Fig. 1:**
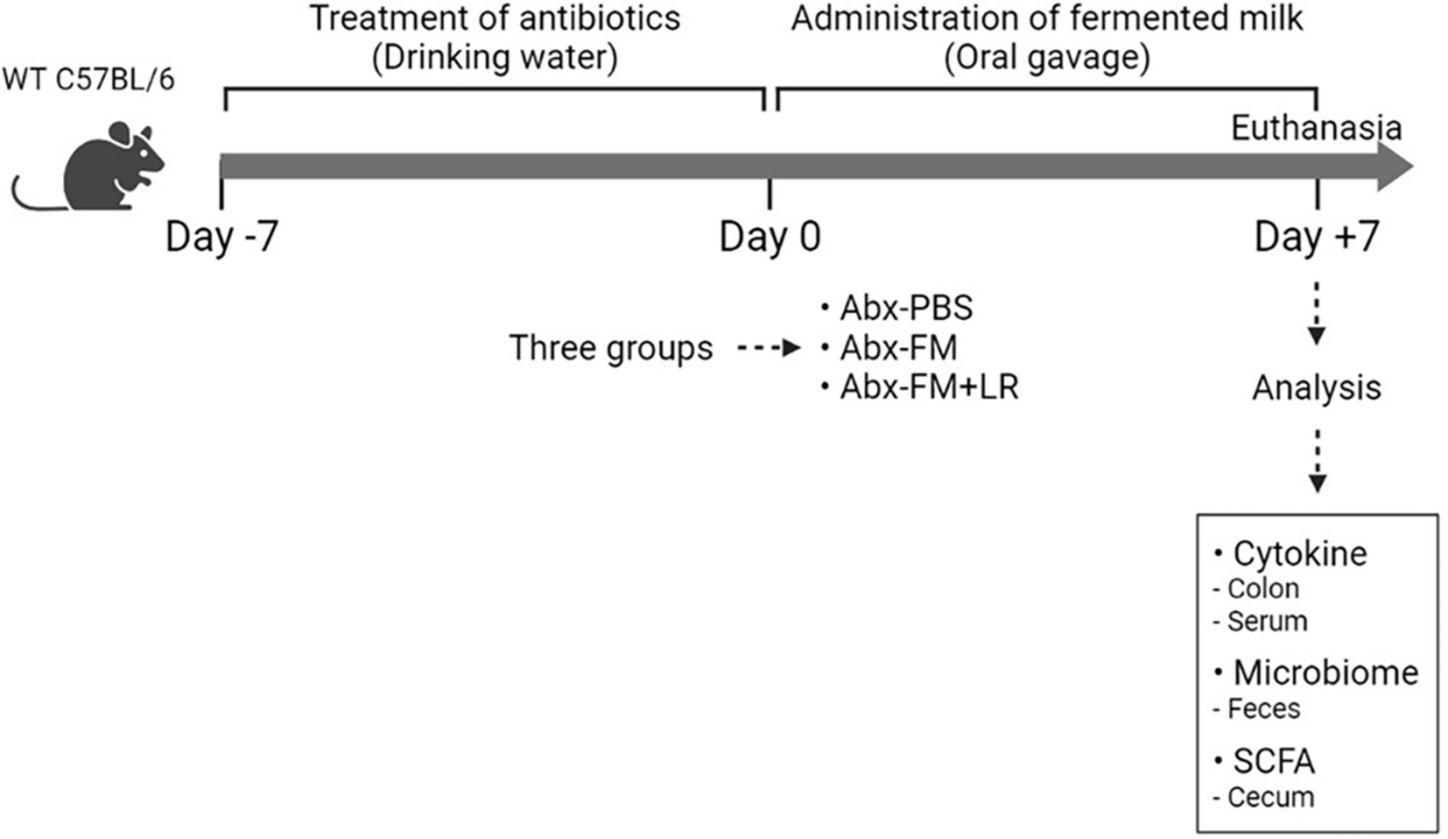
The experimental scheme of this study. A mixture of antibiotics, containing ampicillin, metronidazole, neomycin and vancomycin, was treated to 6 week-old female BALB/c mice via drinking water for 1 week. Then, 200 μl of fermented milk contained 2 × 10^5^ CFUs of *L. rhamnosus* SNUG50430 was administered to mice once daily by oral gavage for 1 week. Colon samples were homogenized and the supernatant was collected after centrifugation at 15,000 ×*g* for 10 min at 4°C. Cytokine levels in the supernatant were measured. The PBS-antibiotics (PBS+Abx)-treated, the fermented milk without *L. rhamnosus* SNUG50430-antibiotics (FM+Abx)–treated, and the fermented milk with *L. rhamnosus* SNUG50430-antibiotics (FM+LR+Abx) group, were designed as each experimental group. Water-treated group was used as a negative control.

The animal model is illustrated in Fig. 1. All experiments including the collection of feces and clinical information were performed in accordance with the relevant guidelines and regulations of the institutional review board of Seoul National University, Republic of Korea (IRB no. 1602/001-001). All animal experimental procedures were approved by the Institutional Animal Care and Use Committee (IACUC: SNU-180104-2-3) of Seoul National University, Republic of Korea. Six week-old female BALB/c mice (Orient Bio Inc., Republic of Korea) were divided into groups of five mice per each experimental condition. Three experimental groups, including the phosphate buffered saline (PBS)-antibiotics (PBS+Abx)-treated, the fermented milk without *L. rhamnosus* SNUG50430-antibiotics (FM+Abx)–treated, and the fermented milk with *L. rhamnosus* SNUG50430-antibiotics (FM+LR+Abx) group, were designed. Mice treated with water comprised the negative control group. For antibiotic treatment, the distilled drinking water including 1 g/l ampicillin (Sigma-Aldrich), 1 g/l metronidazole (Sigma-Aldrich), 1 g/l neomycin (Sigma-Aldrich), and 0.5 g/l vancomycin (Sigma-Aldrich) was supplied to the cages for 1 week [21]. Next, 200 μl fermented milk contained 2 × 10^5^ CFUs of *L. rhamnosus* SNUG50430 was administered to mice once daily by oral gavage for 1 week. After the treatment of fermented milk, the mice were euthanized. Fecal, colon, serum, and cecum samples were collected and stored at −80°C for further analyses.

### Measurement of Cytokines in Colon and Serum Samples

Colon samples were weighed and homogenized in 1× RIPA buffer (Thermo Fisher Scientific, USA) with a Halt Protease Inhibitor Cocktail (Thermo Fisher Scientific) for 5 min using a MM 400 Mixer Mill homogenizer (Retsch, GmbH., Germany), as described previously [22]. The supernatant was collected after centrifugation at 4°C for 10 min at 15,000 ×*g*. Interferon gamma (IFN-γ), interleukin (IL)-2, IL-5, IL-10 and tumor necrosis factor alpha (TNF-α) in the supernatant from colon samples and serum samples were quantified using a LEGENDplex Mouse Th Cytokine Panel 13-plex (Biolegend, USA) following the manufacturer’s instructions.

### Analysis of Fecal Microbiota

DNA from fecal samples was extracted using a QIAamp DNA Stool Mini Kit following the manufacturer’s instructions. (Qiagen, Germany). The V4 region of the 16S rRNA genes was amplified using the universal primers 515F/806R as described previously with some modification [23]. The Polymerase chain reaction (PCR) amplicons were purified using a QIAquick PCR Purification Kit (Qiagen) and quantified using a Quant-iT PicoGreen dsDNA Assay Kit (Thermo Fisher Scientific) following the manufacturer’s instructions. The pooled amplicons were sequenced using a MiSeq platform (Illumina, Inc., USA) as described previously [24]. Sequences for 16S rRNA genes were analyzed using the Quantitative Insights into Microbial Ecology 1.8.0 software (QIIME Development Team; http://qiime.org/) and Greengenes version 13_5 data base (http://greengenes.secondgenome.com)[22]. Sequences were clustered to operational taxonomic units (OTUs) using the OTU picking protocol with at least 97% nucleotide identity. The relative abundances of microbial taxa were calculated using a non-rarefied OTU table. Alpha diversities were described as the Observed species and Sharnon indices and Beta diversities were described as the non-metric multi-dimensional scaling (NMDS) plot, calculated using the Bray-Curtis distance [25]. Phylogenetic investigation of communities by reconstruction of unobserved states (PICRUSt) analyses were performed using Galaxy ver. 2.1.1 (Hutlab; http://huttenhower.org/galaxy) and the Kyoto Encyclopedia of Genes and Genomes pathway database (GenomeNet; https://www.genome.jp/kegg/pathway.html) [22, 26].

### Quantification of SCFAs in Cecum Samples

SCFAs in cecum samples were quantified as described previously with some modification [27]. First, colon samples were homogenized with distilled water and centrifuged for 5 min at 13,000 ×*g*. The supernatant was collected for further analyses. Ethyl ether and 2-methylpentanoic acid (1%) were used as an extraction solvent and an internal standard, respectively. The organic layers from the supernatants were analyzed using an Agilent 7890A gas chromatograph (Agilent Technologies, USA) following conditions as described previously [27]: 1.5 kV for capillary voltage, 600 L/h for desolvation gas flow, 50 L/h for cone gas flow, 170°C for oven temperature, 225°C for a flame ionization detector and an injection port temperature, and nitrogen for a carrier gas. The retention times and peak areas of samples were confirmed using a standard mixture [28].

### Statistical Analysis

Data are expressed as means ± standard error of the mean of three independent experiments. When appropriate, data were analyzed using the Mann-Whitney *U* test or the Kruskal-Wallis one-way analysis of variance with the Dunn’s *post hoc* test. *P*-values (*P*) < 0.05 were considered statistically significant. GraphPad Prism ver. 9.01 (GraphPad Software, Inc., USA) was used for data analyses and visualizations. Spearman's nonparametric correlation coefficients for relative abundances of microbes and cytokine levels were calculated using a GraphPad Prism ver. 9.01 and visualized using the *pheatmap* (ver. 1.0.12) package in R (ver. 3.6.2) (R Core Team, Austria).

## Results

### Effects of Fermented Milk Containing *L. rhamnosus* SNU50430 on Cytokine Levels in Colon and Serum Samples

**Fig. 2:**
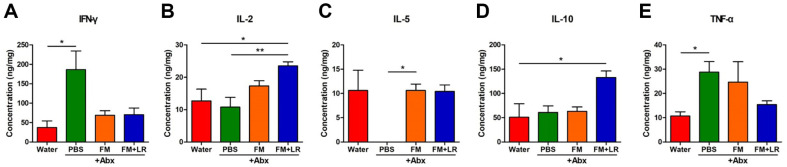
Effects of fermented milk containing *L. rhamnosus* SNUG50430 on cytokine levels in colon samples of antibiotic-treated mice. (**A**) Interferon gamma (IFN-γ), (**B**) Interleukin (**IL**)-2, (**C**) IL-5, (**D**) IL-10, (**E**) Tumor necrosis factor alpha (TNF-α). Data are expressed as the mean ± standard error of the mean (**SEM**) of three independent experiments. Asterisks indicate a statistically significant difference [*P < 0.05; **P < 0.01; Kruskal-Wallis one-way analysis of variance (**ANOVA**) with the Dunn’s *post hoc* test].

Fig. 2 shows effects of fermented milk containing *L. rhamnosus* SNU50430 on cytokine levels in colon samples from mice with antibiotic treatment. PBS+Abx-treated mice showed significant increases in inflammatory cytokine IFN-γ and TNF-α compared to water-treated mice, as a negative control (*P* < 0.05) (Fig. 2A and 2E). Moreover, IL-5 levels in PBS+Abx-treated mice were decreased compared to water-treated mice (Fig. 2C). FM+LR+Abx-treated mice had the highest concentration of pro-inflammatory cytokine IL-2 and anti-inflammatory cytokine IL-10 levels (*P* < 0.05, compared to water-treated mice) (Fig. 2B and 2D).

**Fig. 3:**
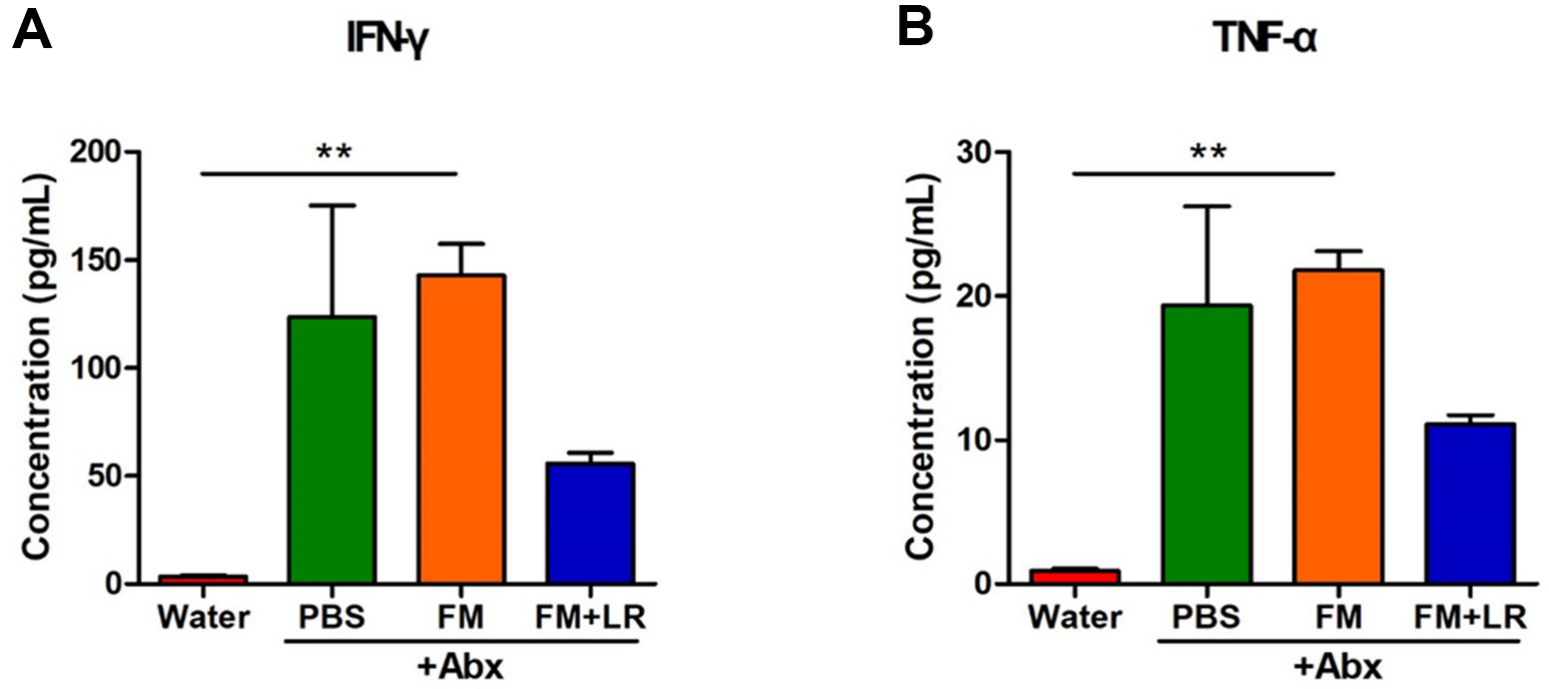
Effects of fermented milk containing *L. rhamnosus* SNUG50430 on cytokine levels in serum samples of antibiotic-treated mice. (**A**) IFN-γ, (**B**) TNF-α. Cytokine levels in the serum collected from mice were measured. Data are expressed as the mean ± SEM of three independent experiments. Asterisks indicate a statistically significant difference (**P < 0.01; Kruskal-Wallis one-way ANOVA with the Dunn’s *post hoc* test).

Mice with antibiotic treatment exhibited an increase in IFN-γ and TNF-α levels in serum compared to water-treated mice (Fig. 3). However, fermented milk containing *L. rhamnosus* SNU50430 showed lower levels of both inflammatory cytokines compared to other antibiotic-treated groups (Fig. 3).

### Effects of Fermented Milk Containing *L. rhamnosus* SNU50430 on Alteration of Fecal Microbiota

**Fig. 4:**
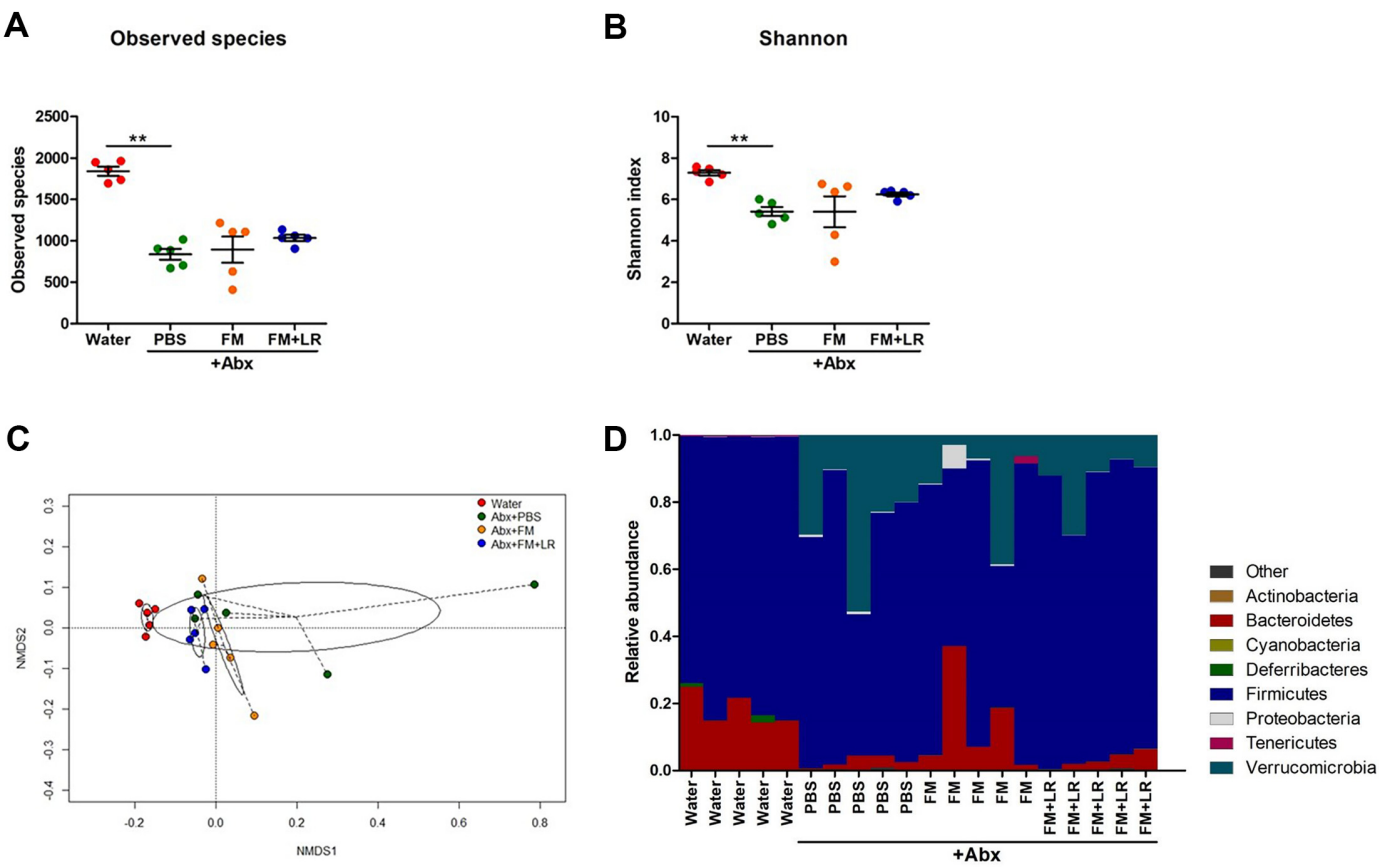
Effects of fermented milk containing *L. rhamnosus* SNUG50430 on fecal microbiota in antibiotictreated mice. (**A**) Observed species and (**B**) Shannon indices of each experimental group for Alpha-diversity, (**C**) Non-metric multi-dimensional scaling (**NMDS**) plot with Bray-Curtis distances for experimental groups, (**D**) Comparisons of microbial taxa of experimental group at phylum level. Data are expressed as the mean ± SEM of three independent experiments. Asterisks indicate a statistically significant difference [**P < 0.01; Kruskal-Wallis one-way ANOVA with the Dunn’s *post hoc* test].

Fig. 4 summarizes the effects of fermented milk on the fecal microbiota of antibiotic-treated mice. Compared to water-treated mice, bacterial diversities for PBS+Abx-treated mice were significantly decreased (*P* < 0.01) (Fig. 4A and 4B). Microbial communities in feces for both FM+Abx-treated and FM+LR+Abx-treated mice showed abundant diversities and distinctively clustered compared to PBS+Abx-treated mice (Fig. 4A–4C). The dominant bacterial communities in feces of water-treated mice are phylum Firmicutes and Bacteroidetes (Fig. 4D). However, in feces of PBS+Abx-treated mice, phylum Firmicutes and Bacteroidetes were less abundant whereas phylum Verrucomicrobia was significantly increased (Fig. 4D). The abundance of phylum Firmicutes was restored and the decrease in phylum Verrucomicrobia and Proteobacteria were discovered in FM+LR+Abx-treated mice compared to PBS+Abx-treated mice (Fig. 4D).

**Fig. 5:**
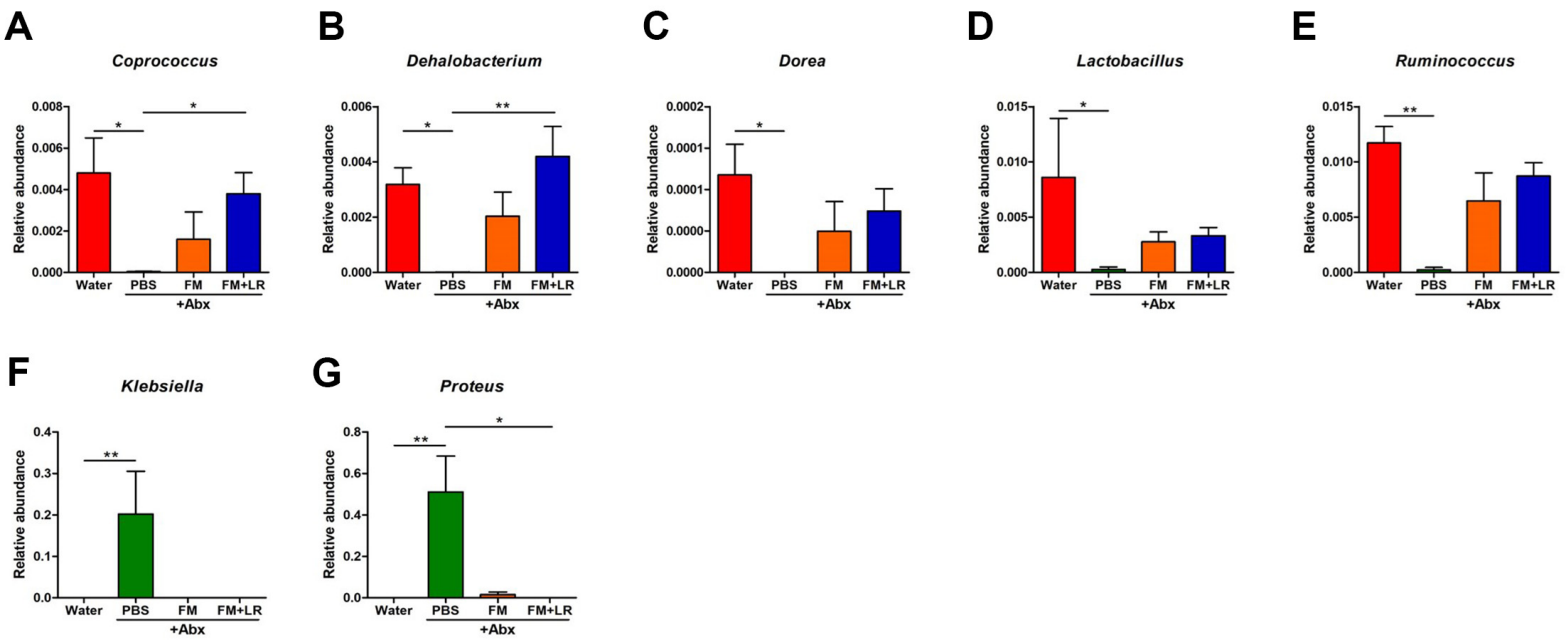
Relative abundances in microbial genera among experimental groups. (**A**) Genus *Coprococcus*, (**B**) Genus *Dehalobacterium*, (**C**) Genus *Dorea*, (**D**) Genus *Lactobacillus*, (**E**) Genus *Ruminococcus*, (**F**) Genus *Klebsiella*, (**G**) Genus *Proteus*. Data are expressed as the mean ± SEM. Asterisks indicate a statistically significant difference (*P < 0.05; **P < 0.01; Kruskal-Wallis one-way ANOVA with the Dunn’s *post hoc* test).

The relative abundance of genus *Coprococcus* (*P* < 0.05) and *Dehalobacterium* (*P* < 0.01) were significantly increased in FM+LR+Abx-treated mice compared to PBS+Abx-treated mice (Fig. 5A and 5B). The relative abundances of genus *Dorea*, *Lactobacillus*, and *Ruminococcus* showed upward tendencies in both FM+Abx-treated and FM+LR+Abx-treated mice compared to PBS+Abx-treated mice (Fig. 5C–5E). The highest relative abundance of genus *Klebsiella* (*P* < 0.01, compared to water-treated mice) and *Proteus* were discovered in PBS+Abx-treated mice (*P* < 0.01 compared to water-treated mice and *P* < 0.05 compared to FM+LR+Abx-treated mice, respectively) (Fig. 5F and 5G).

### Correlations between Relative Abundances of Microbial Taxa and Cytokine Levels in Mice

**Fig. 6:**
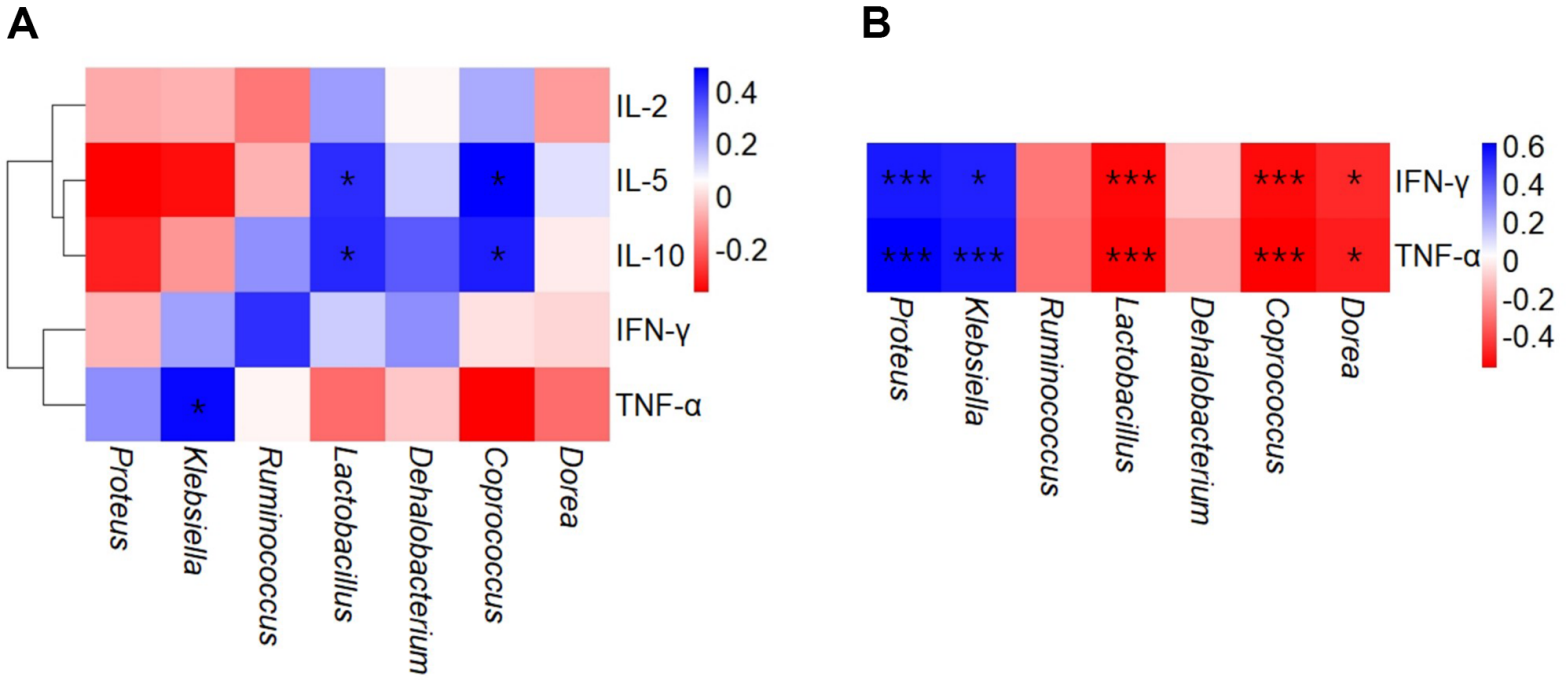
Spearman's correlations between relative abundances of microbial genera and cytokine levels in mice. (**A**) Colon samples, (**B**) Serum samples. Colors indicate the degrees of correlation. Asterisks indicate statistical significance (*P < 0.05; ***P < 0.001).

Fig. 6 shows correlations between relative abundances of microbial taxa and cytokine levels in antibiotic-treated mice. In colon samples, relative abundance of genus *Coprococcus* and *Lactobacillus* had strong positive correlations with cytokine IL-5 and IL-10 levels (*P* < 0.05) (Fig. 6A). The relative abundance of genus *Klebsiella* was positively correlated with TNF-α levels (*P* < 0.05) (Fig. 6A).

Relative abundances of genus *Coprococcus* and *Lactobacillus* had significant negative correlation with serum IFN-γ and TNF-α levels (*P* < 0.001) (Fig. 6B). On the other hand, the relative abundance of genus *Klebsiella* (*P* < 0.05) and *Proteus* (*P* < 0.001) were positively correlated with serum IFN-γ and TNF-α levels (Fig. 6B).

### Effects of SCFA Concentrations and Butyrate Metabolism in Mice

**Fig. 7:**
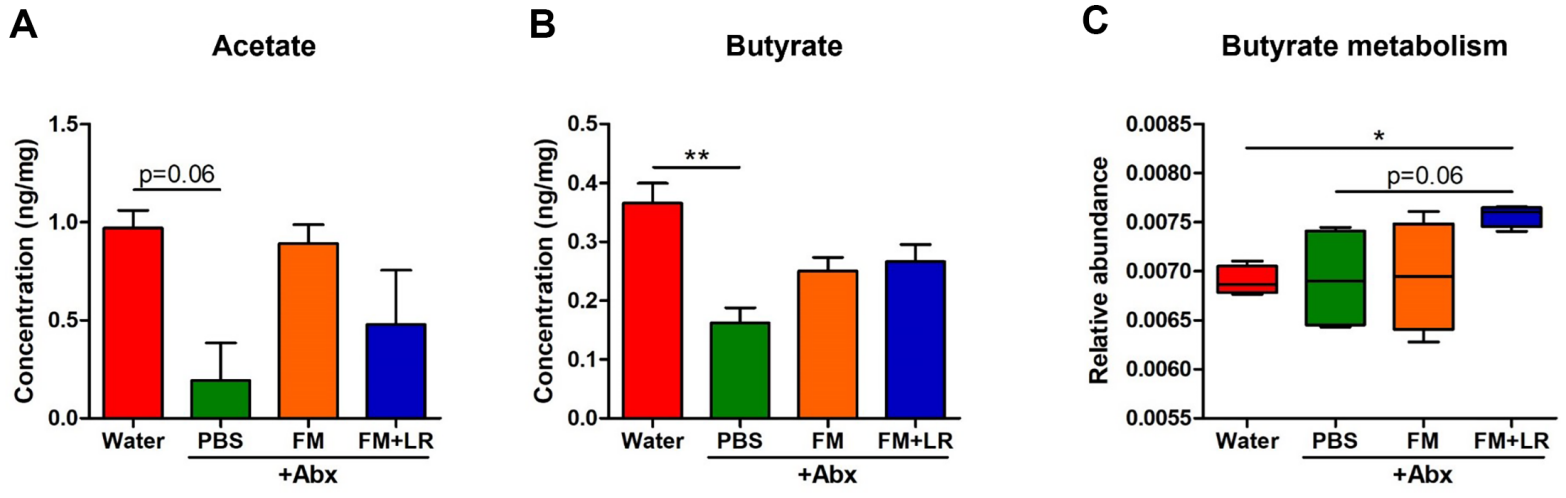
Alterations in short-chain fatty acid (SCFA) concentrations and butyrate metabolism according to the phylogenetic investigation of communities by reconstruction of unobserved state (PICRUSt) analysis in antibiotic-treated mice fed fermented milk containing *L. rhamnosus* SNUG50430. (**A**) Acetate concentration, (**B**) Butyrate concentration, (**C**) PICRUSt analysis for butyrate metabolism. SCFAs in samples were measured using an Agilent 7890A gas chromatograph. Data are expressed as the mean ± SEM. Asterisks indicate a statistically significant difference (*P < 0.05; **P < 0.01; Kruskal-Wallis one-way ANOVA with the Dunn’s *post hoc* test for SCFA concentrations in experimental groups and Mann-Whitney U test for PICRUSt analysis).

Fig. 7 exhibits alterations of SCFA concentrations in cecum samples on mice with antibiotic treatment. PBS+Abx-treated mice showed the lowest concentration of acetate and butyrate (Fig. 7A and 7B). Fermented milk showed increases in SCFA concentrations of FM+LR+Abx-treated or FM+Abx-treated mice (Fig. 7A and 7B). Fecal microbiota of FM+LR+Abx-treated mice were highly enriched with butyrate metabolism pathway compared to water-treated mice (*P* < 0.05) and PBS+Abx-treated mice (*P* = 0.06, marginally significant) (Fig. 7C).

## Discussion

In this study, we evaluated health effects of fermented milk containing *L. rhamnosus* SNU50430 in antibiotic-treated mice. IFN-γ and TNF-α levels were decreased in FM+LR+Abx-treated mice compared to PBS+Abx-treated mice (Figs. 2 and 3). Moreover, the addition of *L. rhamnosus* SNU50430 to fermented milk enhanced the levels of the pro-inflammatory cytokine IL-2, which is an important CD25 activator on regulatory T cells (Tregs)[29], and the anti-inflamatory cytokine IL-10 in colon samples (Fig. 2). Fermented milk can reduce the levels of inflammatory cytokines of host [30, 31]. Moreover, *L. rhamnosus* strains, such as *L. rhamnosus* GG, have been shown to exert strong immunomodulation effects in various experimental models [32-34]. Therefore, *L. rhamnosus* SNU50430 can be applied to fermented milk for enhancing anti-inflammatory effects in hosts on antibiotic treatment.

Compared to the PBS+Abx-treated mice, fecal microbiota of FM+LR+Abx-treated mice showed upward tendencies in restoration of bacterial diversities (Fig. 4A and 4B) and clustered distinctively among the other groups (Fig. 4C). Gut microbiota dysbiosis is one of the major side-effects of antibiotic treatment, which can affect adverse physiological activities of host [35, 36]. Probiotics in fermented milk can restore bacterial diversities and enrich beneficial microorganisms in gut [37, 38]. Positive effects of *L. rhamnosus* strains in gut microbiota also have been reported in various studies [31, 39, 40], therefore, *L. rhamnosus* SNU50430 had important roles for gut microbiota in antibiotic-treated mice. Especially, the abundance of phylum Verrucomicrobia and Proteobacteria was clearly decreased in FM+LR+Abx-treated mice compared to the antibiotic-treated mice (Fig. 4D). Phylum Verrucomicrobia shows strong resistances in a variety of antibiotics [25] and phylum Proteobacteria has been reported to exert adverse health effects [41]. Genus *Klebsiella* and *Proteus*, which are important pathobionts in phylum Proteobacteria, were significantly increased in PBS+Abx-treated mice (Fig. 5F and 5G) [42]. Moreover, our fermented milk restored relative abundances of beneficial microorganisms, including genus *Lactobacillus* and *Ruminococcus*, in gut [43, 44], indicating that the fermented milk has positive effects on host gut microbiota, which can be distort significantly due to antibiotic treatment (Fig. 5). However, further longitudinal studies should be performed to explore the effects of *L. rhamnosus* SNU50430 in fermented milk on host gut microbiota fully.

The relative abundance of genus *Coprococcus*, *Dorea*, and *Lactobacillus* was positively correlated with the levels of anti-inflammatory cytokine IL-5 and IL-10 in colon samples and had significant negative correlation with the levels of inflammatory cytokines IFN-γ and TNF-α in serum samples in mice (Fig. 6A and 6B). Our results indicated that significant negative correlations between abundances in beneficial microorganisms in gut, which were caused by treatment of fermented milk containing *L. rhamnosus* SNU50430, and inflammatory responses on host due to antibiotic treatment were discovered. Gut microbiota can modulate gut immunity and maintain gut immune homeostasis [45, 46]. Therefore, *L. rhamnosus* SNU50430 can enhance beneficial effects of fermented milk to improve damaged gut microbial composition and host immunity due to antibiotic treatment.

Our results confirmed that fermented milk containing *L. rhamnosus* SNU50430 increased proportions of major genera in family *Lachnospiraceae*, such as genus *Coprococcus*, *Dorea*, and *Ruminococcus* in antibiotic treated mice (Fig. 5A, 5C, and 5E). Multiple genera in family *Lachnospiraceae* are SCFA-producing bacteria [47]. SCFAs have been known to promote anti-inflammatory effects on host with amelioration of mucosal inflammation and stimulation of regulatory T cells [48]. Therefore, we assumed that changes in gut microbiota caused by fermented milk can affect both SCFA concentrations in gut and their related metabolic pathways (Fig. 7). However, many other metabolites are produced by gut microbiota which can influence metabolisms and immunities of host. Therefore, further studies are needed to investigate the relationships among other gut microbiota metabolites with host immune parameters due to fermented milk containing *L. rhamnosus* SNU50430 in host with antibiotic treatment.

## Conclusion

*L. rhamnosus* SNU50430 significantly enhanced health effects of fermented milk in antibiotic-treated mice. Especially, the fermented milk with *L. rhamnosus* SNU50430 increased the levels of anti-inflammatory cytokines in both colon and serum and restored of damaged gut microbiota. Therefore, fermented milk with *L. rhamnosus* SNU50430 shows potential for ameliorating the adverse health effects of antibiotics.
